# Molecular phylogeny and historical biogeography of marine palaemonid shrimps (Palaemonidae: *Palaemonella*–*Cuapetes* group)

**DOI:** 10.1038/s41598-022-19372-5

**Published:** 2022-09-08

**Authors:** Pavlína Frolová, Ivona Horká, Zdeněk Ďuriš

**Affiliations:** Department of Biology and Ecology, Faculty of Science, University of Ostrava, Chittussiho 10, 710 00 Ostrava, Czech Republic

**Keywords:** Ecology, Evolution, Zoology

## Abstract

Palaemonidae is the most speciose shrimp family within the infraorder Caridea, composed predominately of freshwater species and marine symbiotic species. The subject of this study is a clade of mainly free-living marine taxa representing a basally separated lineage from most of the symbiotic marine palaemonid genera. Phylogenetic and biogeographic relationships were explored by analysing sequence data from two mitochondrial and four nuclear markers. Maximum likelihood and Bayesian analyses, based on sequences from 52 species of 11 genera, provided similar tree topologies revealing the genera *Palaemonella*, *Cuapetes* and *Eupontonia* as non-monophyletic groups. Divergence time and S-DIVA analyses reveals that the focal clade originated during the Late Cretaceous in the Paleotethys region respective to the present Indo-West Pacific area, a minor part of which spread out to the eastern Pacific during the Paleocene, followed by further migration into the Atlantic (before the closure of the Panama Isthmus). The ancestral state reconstruction of host associations revealed eight independent symbiotic lineages originating from free-living ancestors, entering primary symbioses. The first associations with Cnidaria are estimated to have evolved in the Eocene. This study points to the need of taxonomic revisions of the non-monophyletic genera concerned.

## Introduction

The family Palaemonidae, including presently more than 1200 species in 160 genera, represents the largest family of the infraorder Caridea^1^. Representatives of palaemonid shrimp occur in marine and freshwater environments in tropical to temperate regions around the world, with the highest abundance and diversity of marine species in the Indo-West Pacific (IWP) biogeographic area^2^.

Palaemonid shrimps are free-living or occur in association with a variety of different hosts to benefit from nutrient sources, shelter and reproductive opportunities. According to previous studies, about 70% of them participate in symbiotic interactions. Symbiotic relations can be distinguished as mutualistic, commensal, or parasitic, depending on both the costs and benefits of the coexistence and the level of dependency on the host (facultative/obligatory)^3–5^.

Recent molecular phylogenetic studies have shown the family Palaemonidae (in its traditional concept)^6^ to be polyphyletic, mainly due to the families Hymenoceridae and Gnathophyllidae nested inside (e.g.;^4,7–9^), and the subfamilies Pontoniinae (mainly symbiotic) and Palaemoninae (free-living, mainly freshwater) proved to be polyphyletic^9^. Consequently, De Grave et al.^9^ formally invalidated the subfamilial concept of the family and synonymised Gnathophyllidae, Hymenoceridae, and Pontoniinae with Palaemonidae. More recently, Anchistioididae has also been incorporated into the family^10^.

Among the traditional pontoniine taxa, the molecular study by Chow et al.^10^ highlighted two sister lineages—the minor one formed by mostly free-living species of the genera around *Cuapetes* and *Palaemonella* (called Pon-I in that study) and the major one comprising almost exclusively symbiotic taxa (Pon-II). For the purposes of this study, we follow to the latter study to mark those groups Pon-I and Pon-II.

**Figure 1:**
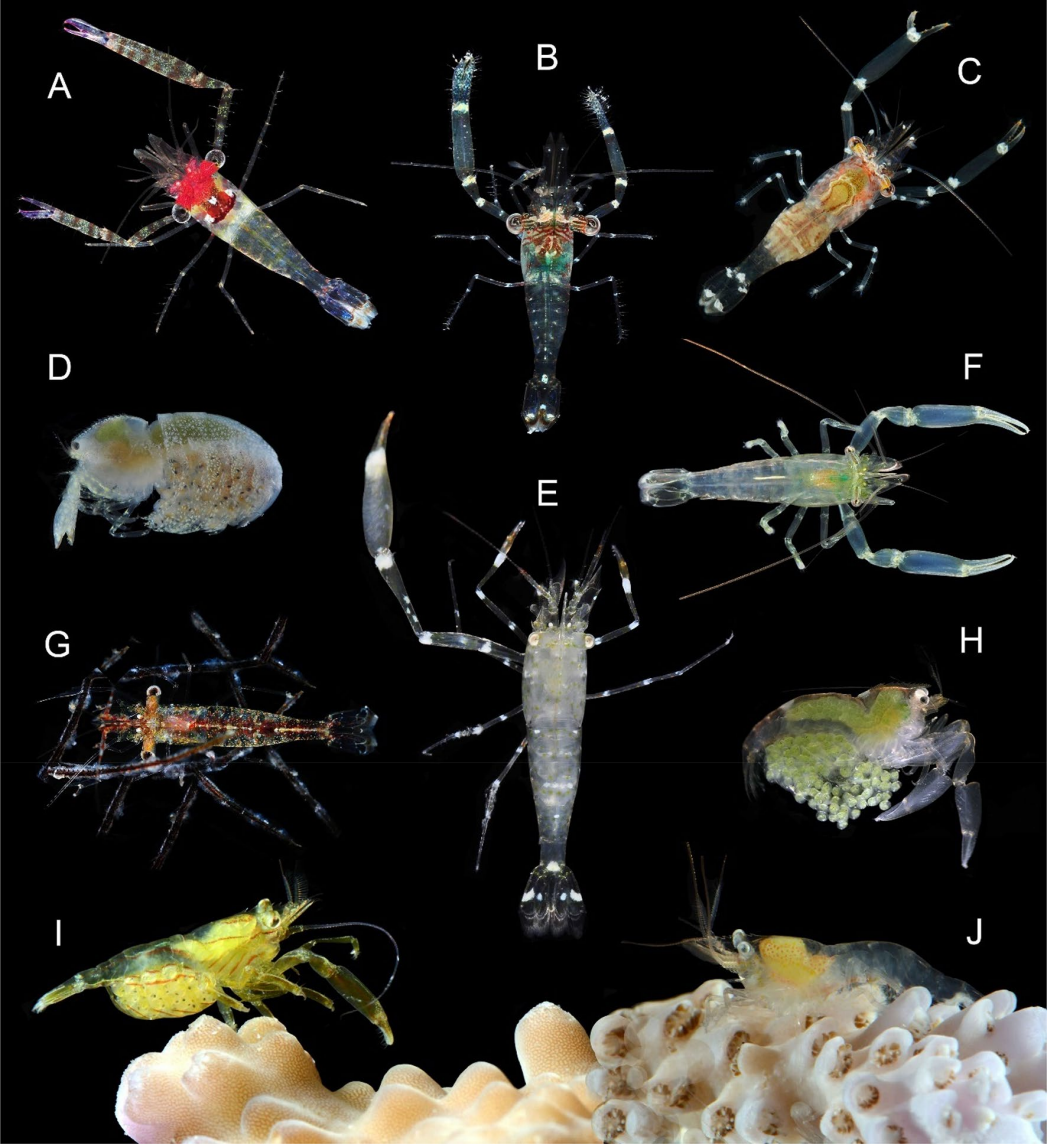
Photographs of symbiotic palaemonid shrimp. (**A**) *Exoclimenella maldivensis*; (**B**) *Periclimenella spinifera*; (**C**) *Cuapetes amymone*; (**D**) *Anapontonia denticauda*; (**E**) *Palaemonella aliska*; (**F**) *Harpilius lutescens*; (**G**) *Cuapetes nilandensis*; (**H**) *Ischnopontonia lophos*; (**I**) *Philarius imperialis*; (**J**) *Vir orientalis*. Author of photographs: Zdeněk Ďuriš.

The target subject of the present study, respective to the Pon-I group highlighted by Chow et al.^10^, currently comprises 85 predominantly free-living species belonging to 11 genera (Fig. 1) from which the most species-rich are *Cuapetes* (29 spp.) and *Palaemonella* (23 spp.). All these shrimps form an independent clade, basally separated from the major clade of the symbiotic ‘pontoniines’ (Pon-II)^10^, equally recovered in the molecular studies of Kou et al.^7^, Horká et al.^4^, and Chow et al.^5^. While almost all species of this clade (80 spp.) live in the IWP, six are known outside from that area—in the eastern Pacific (EP; 2 spp.), the western Atlantic (2 spp.), and in the eastern Atlantic (2 spp., a further two Lessepsian migrant species, are not included)^11^. A minority of the Pon-I shrimps are known for their symbiotic relationships. Mostly, they are ectosymbionts of cnidarians and in one case of crinoids^12,13^, but the inquilinism, i.e., living in burrows constructed by alpheids, opistognathid jawfish, or echiurids, is known in three species^5,14,15^.

The historical biogeography and evolution of palaemonid shrimp were determined by four major vicariant events, i.e., (1) the opening of the central Atlantic Ocean (∼ 170 Myr), (2) the formation of the East Pacific Barrier (∼ 65 Myr), (3) the final closure of the Isthmus of Panama (∼ 14–2.8 Myr), and (4) the closure of the Tethys seaway between the Indian and Atlantic Oceans (∼ 12 Myr) ^16–20^. The first two are soft vicariance events (barriers) that do not represent a direct physical barrier with a permanent impact on the dispersal capabilities of marine taxa. The other two are hard barriers caused by the formation of land bridges which physically divide and completely genetically isolate marine populations^17,21^. This study aimed to reconstruct the molecular phylogeny of the Pon-I group of palaemonid shrimps species based on a six-marker molecular analysis of the major diversity of the Pon-I shrimps, covering most taxa (~ 60%) from the IWP, most species known from outside the IWP, and almost all known taxa entering symbiotic relationships. The further objectives of this study, based on ancestral state reconstruction and divergence time estimates, are to provide insights into their historical biogeography and the phenomenon of the primary symbiotic relationships established within the target group.

We hypothesize that: (1) the present group of these palaemonid shrimps originated in the IWP (as part of the past Tethys Ocean), and subsequently dispersed through the eastern Pacific to the western Atlantic, and then to the eastern Atlantic; (2) on the general free-living background, the co-existence with another animals was established multiply times within the group via primary symbioses; and, (3) the most primordial symbiotic relations within the present group were with cnidarian hosts.

## Results

For this study, 52 species represent the studied Pon-I group, and three species of the related palaemonid genera were selected as outgroup. Representatives of the main tropical regions, i.e. the Indo-West Pacific biogeographic area (IWP, 50 from 81 known spp.) and the eastern Pacific (EP, 2/2), western Atlantic (WA, 2/2), and the eastern Atlantic (EA, 1/2 sp.) subregions of the Atlanto-East Pacific biogeographic area, were analysed.

### Phylogeny

**Figure 2:**
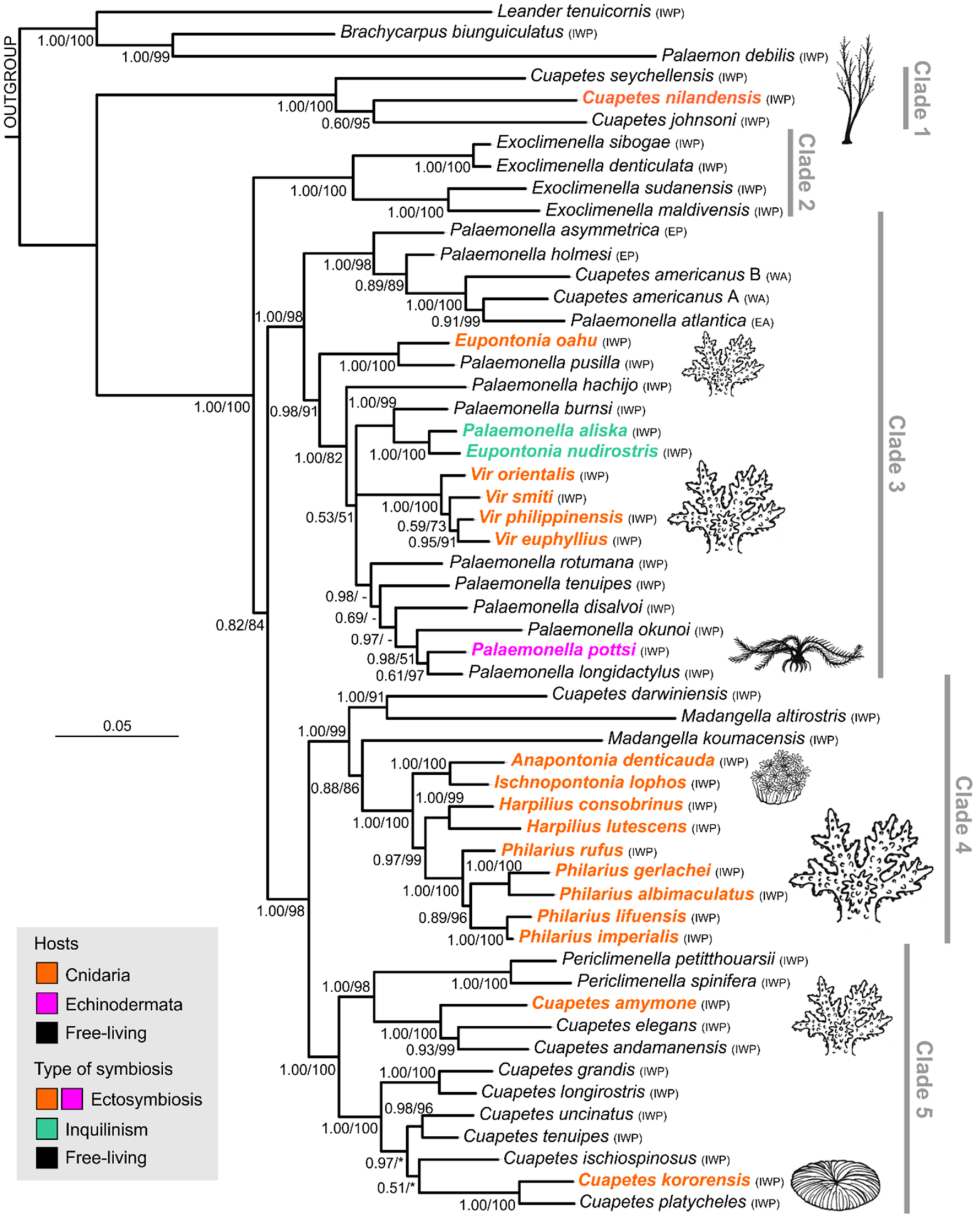
Phylogenetic tree of symbiotic shrimp taxa resolved by Bayesian inference based on the combined dataset for six genes (COI, 16S, H3, 18S, Enol, NaK). Ultrafast bootstrap (UFB) support is expressed as a percentage. Dash (–) indicates UFB values < 50; asterisk (*) indicates different topology of Maximum Likelihood tree. Major clades and host affiliations are highlighted. Abbreviations of areas: *IWP* Indo-West Pacific, *EP* eastern Pacific, *WA* western Atlantic, *EA* eastern Atlantic.

The multigene molecular analysis of the six gene markers (16S, COI, H3, 18S, Enol, NaK) comprises 3379 characters. The resulting trees based separately on the ML and BI methods produced identical general topologies with comparable support values (Fig. 2). All main clades (1–5) are well-supported (PP and UFBoot ≥ 95), just the mutual relationships among the Clade 3 and the Clades 4–5 remain unresolved due to low basal support. Clade 1, most basally separated from the main diversity of the examined Pon-I taxa, is formed by three IWP species of the genus *Cuapetes*. Clade 2 contains all four currently known species of the genus *Exoclimenella*. Clade 3 is predominantly composed of species of the genus *Palaemonella*; this clade is further separated into two geographically delineated subclades—one is composed of American (EP) or Atlantic (WA, EA) species; the other subclade comprises IWP species of the genus, but includes also *Vir* and *Eupontonia* species. Clade 4 contains two sister-positioned subclades of IWP taxa; the first supported subclade contains the species *C. darwiniensis* and *Madangella altirostris*; the second subclade is not supported basally due to the unresolved position of *M. koumacensis*; the remaining, major, part of the clade is constituted by a compact, well-supported clade of the genera *Anapontonia*, *Ischnopontonia*, *Harpilius* and *Philarius*. Clade 5 contains the remaining main diversity of the genus *Cuapetes*, with an inclusion of two *Periclimenella* species.

### Evolution of host associations

**Figure 3:**
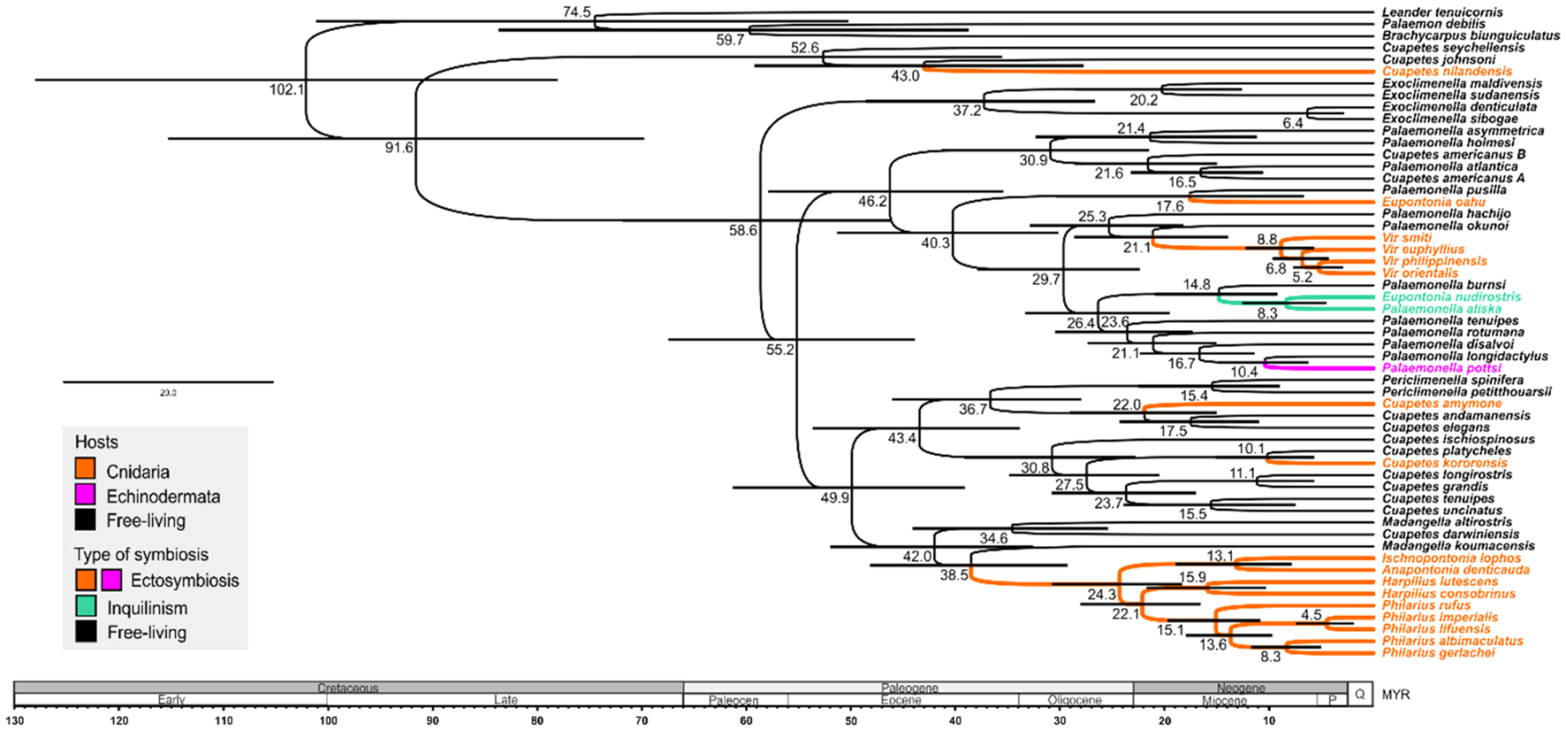
Divergence time estimates based on BEAST analysis using mutation rates and ancestral state reconstruction of host associations. The bars represent the highest posterior density (95%).

In the present Ancestral State Reconstruction analysis (ASR; Fig. 3), the species with a symbiotic lifestyle (Fig. 2) are nested in eight separate evolutionary lineages highlighted as five single-species terminals, one multispecies, and two multigeneric compositions. According to the ASR, all symbiotic lineages developed independently from free-living ancestors, and are currently nested separately among free-living taxa. For the Pon-I group, the divergence time analysis assumes that the association with corals was the first to form (Fig. 3). The oldest symbiotic interaction is indicated as the association of *C. nilandensis* with antipatharian corals dated around 43 Myr (27.8–59.2), shortly followed by the association with scleractinian corals (lineages of genera *Anapontonia*, *Harpilius*, *Ischnopontonia* and *Philarius*) dated at 38.5 Myr (29.3–48.1), and the genus *Vir*, dated at 21.1 Myr (14.0–28.6). The association with crinoids (*P. pottsi*) developed later and was dated at 10.4 Myr (6.3–14.8). Furthermore, the inquilinistic forms (*P. aliska* and *E. nudirostris*) originated at 14.8 Myr (9.2–20.8).

### Biogeographical analyses and molecular dating

**Figure 4:**
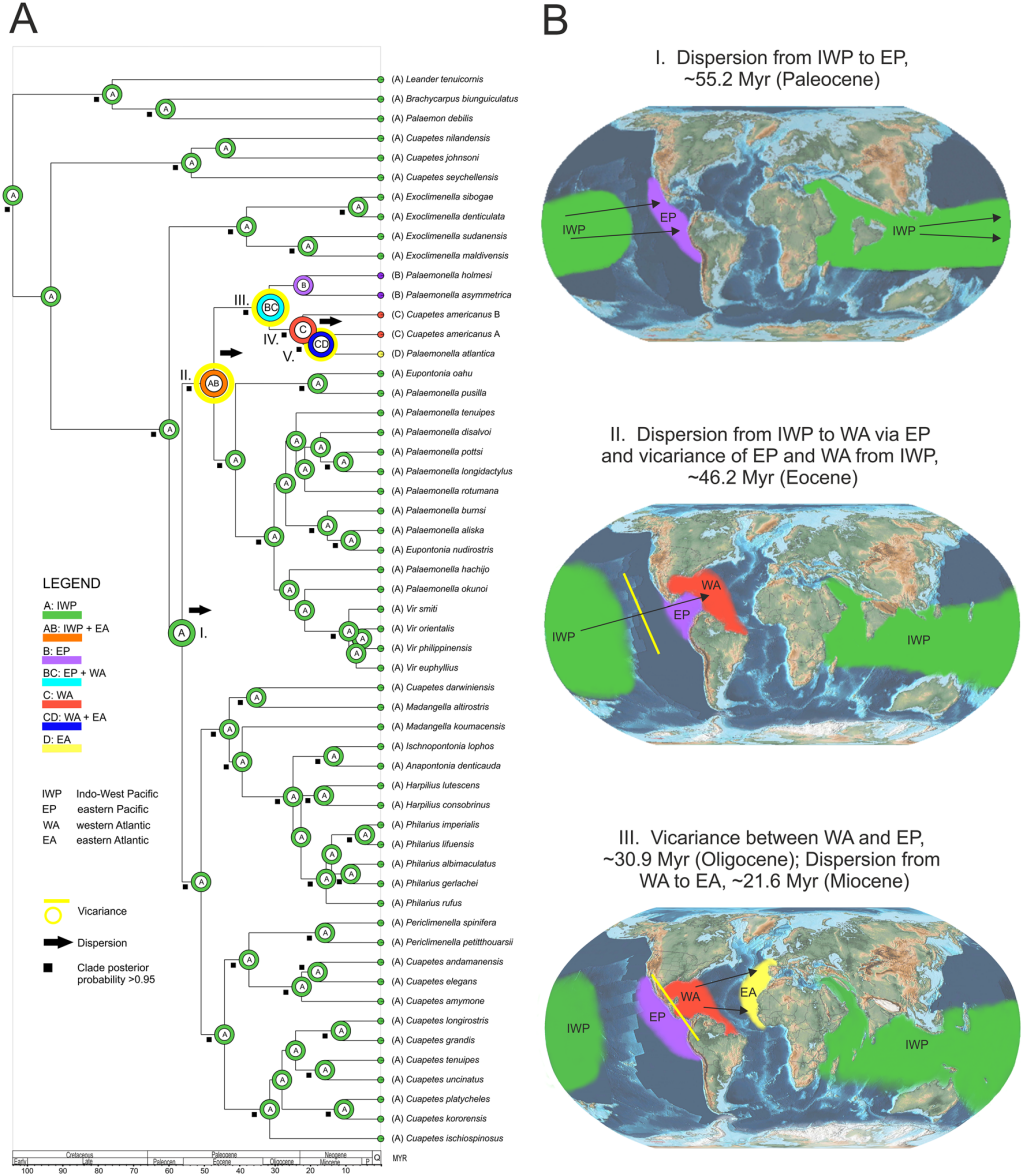
Biogeographic history of palaemonid shrimp (Pon-I group). (**A**) Results from S-DIVA analysis (performed in RASP) of ancestral distributions at each node of the phylogeny. Each node shows the most likely ancestral area for the node. Tree topology is based on BEAST analysis. Geographical areas are coded with letters and colours. (**B**) Paleogeographic maps showing the vicariant and dispersal events. Maps were created using GPlates v2.3 software (http://www.earthbyte.org/paleomap-paleoatlas-for-gplates/).

The results of the statistical dispersal-vicariance analysis (S-DIVA; Fig. 4) allow us to estimate the ancestor of the Pon-I group originating in the bounds of the present-day IWP (Fig. 4A: nodes A) of the past Tethys Ocean, dated to the Late Cretaceous at 91.6 Myr (69.8–115.3). The first dispersal event (node I; probability of ancestral range at node, *P* = 0.528) was recorded from the IWP (A) to the EP (B) dated to the Paleocene at 55.2 Myr (43.9–67.4). Furthermore, the ancestral region for the genus *Palaemonella* (node II; *P* = 0.483) was somewhat equivocal, but a composition area including the IWP + EP (AB) was slightly favoured (52%) and dated at 46.2 Myr (35.4–57.8). The latter node suggests a dispersal event of some *Palaemonella* species into WA (C) and a vicariance event separating the EP + WA (BC) clade from the rest of the genus in the IWP (A). Another vicariance event (node III; *P* = 0.905) separated the WA (C) clade from the EP (B) clade, dated to the Oligocene at 30.9 Myr (21.5–41.3). The ancestral region for the Atlantic species of the genus *Palaemonella* (node IV; *P* = 0.990) was optimised in WA (C), with a dispersal event into EA (D) that was dated at 21.6 Myr (15.0–28.6), followed by a vicariance event (node V; *P* = 1.000) separating these two areas. The separation of the clade EA (D) dates to 16.5 Myr (10.6–23.2).

## Discussion

Phylogenetic relationships inside the family Palaemonidae remain unresolved, despite being frequently discussed in recent publications^9,10^. Nevertheless, the last published study^5^ presented the main lineages of the family as well supported. Among those, the studied Pon-I group of predominantly free-living taxa is basal-positioned to the remaining genera of the former subfamily Pontoniinae, usually more specialised and associated with a wide range of hosts. The basal separation of the symbiotic genera led some authors to consider the assemblage, following Bruce^22^, to be a primitive group, or descendants of such^7,23^. Additionally, Gan et al.^8^ suggested that the taxa of the Pon-I group might be direct descendants of the ancestors of the former subfamily Pontoniinae, sharing the main plesiomorphies appearing frequently in former palaemonine taxa, e.g., the genera *Brachycarpus*, *Leptocarpus*, *Macrobrachium*, or *Palaemon*. The median process on the fourth thoracic sternite can be considered a plesiomorphic feature; indeed, it is a common symplesiomorphy of all Pon-I taxa, including *Ischnopontonia* and *Anapontonia*, for which the process was formerly reported as missing^24^ (its presence was confirmed in present examined specimens). In addition to that, the mandibular palp occurring in the genera *Exoclimenella*, *Eupontonia*, *Palaemonella*, and *Vir*^25^, or the presence of two arthrobranchs on the third maxilliped in *Exoclimenella*^26^, can also be considered plesiomorphic features.

The Pon-I group’s internal relations have been unclear until now due to lower generic and species coverage in previous studies^4,5,8^. The present analysis based on a six-marker molecular dataset allows a deeper insight into the phylogenetic relationships of the study group involving all 11 currently recognised genera, and represented by 52 species, i.e. about 60% of the overall known species diversity of the group. The results provide a strong support for the monophyly and/or taxonomic validity of the current genera *Exoclimenella*, *Anapontonia*, *Ischnopontonia*, and suggest the monophyly of genera *Harpilius* and *Philarius*. Moreover, the results reveal non-monophyly of the most speciose genera *Palaemonella* and *Cuapetes*, as well as the species-poor *Eupontonia.* The genus *Palaemonella* was found to be paraphyletic owing to the nested species of the genera *Eupontonia* and *Vir*, which all share a common synapomorphy, the presence of the mandibular palp (mentioned above). Such conclusion was expressed also in the study of Chow et al.^5^.

The present phylogenetic analysis confirmed that the genus *Cuapetes* is not monophyletic, as found to a lesser extent, in a few previous molecular studies ^4,5,23^. In this study, the genus *Cuapetes* was recovered in four separate genetic lineages. The type species *C. nilandensis* is nested in the Clade 1 along with *C. johnsoni* and *C. seychellensis*. This phylogenetic finding is in line with the study of Marin and Sinelnikov^27^, who indicated morphological differences between two of the above-mentioned species and most of the remaining species of the genus (respective of the present Clade 5, also covering *C. grandis*, the type species of the ex-genus *Kemponia*), and questioned the validity of the two latter generic names. The further genetic lineage is shown by the position of *C. americanus* nested in the eastern Pacific—Atlantic branch of the genus *Palaemonella* (Clade 3). This result is also supported by recent phylogenetic studies suggesting the different systematic positions of this species^4,5,10^. Due to the lack of the mandibular palp, the species had been properly, but evidently incorrectly, assigned to the genus *Cuapetes*. The fourth genetic lineage is shown by a separate position of *C. darwiniensis* in the Clade 4 as the sister species of *Madangella altirostris*.

The remaining majority of the *Cuapetes* species (Clade 5) are heterogeneous due to comprising also representatives of the genus *Periclimenella*. Ďuriš and Bruce^26^ hypothesised, based on morphological traits (mainly the unique shape of the first pereiopod chelae and the distinctly asymmetrical and specific second pereiopods), that the genera *Exoclimenella* and *Periclimenella* are closely related. Nevertheless, the present study revealed *Periclimenella* as a part of the genus *Cuapetes*. This result was previously supported in the molecular study by Horká et al.^4^ and weakly supported by Kou et al.^23^.

Fossil records of palaemonid shrimps are rare due to their aquatic habit and poorly calcified exoskeletons. Only a few palaemonid representatives are known compared to many extant taxa; the oldest fossil records contain only genera from the previous subfamily Palaemoninae from the Lower Cretaceous (middle Albian, 100 Myr)^28^. For this reason, we used the known mutation rate of mitochondrial gene (16S rRNA) for dating rather than fossil records.

The present inferred phylogeny and ancestral analysis indicate multiple formations of primary symbioses within the clades dominated by free-living relatives, as shown by previous molecular analyses^4,5^. Our results revealed eight independent lineages within the Pon-I group that evolved from free-living ancestors (Fig. 3). Free-living palaemonids (*Exoclimenella*, *Palaemonella*, *Cuapetes*; Fig. 2) are characterised by an elongate body shape with a dentate rostrum, slender, long, a/symmetrical chelipeds and slender ambulatory pereiopods with simple dactyli. Their carapace might bear the full complement of teeth (i.e., supraorbital, antennal, hepatic, epigastric)^25^. Primary symbiotic forms do not fundamentally differ morphologically from free-living ancestors. Their adaptations to the host affiliation have mainly manifested by changes in body shape, colouration, and the reduction of carapace ornamentation. Their hosts belong to different invertebrate phyla, including Cnidaria (mainly Scleractinia and Antipatharia^22^) and Echinodermata (Crinoidea^29^) in ectosymbiotic forms, but also to spoon worms (Echiura), burrowing Crustacea (alpheid shrimps), and/or gobiid fishes^15^, in inquilinistic forms.

While scleractinian corals were hypothesised as the primary hosts of palaemonid shrimp commensalism^7^, our results revealed the antipatharian association as possibly the earlier one among the Pon-I shrimps. That association was established via a single speciation act at approximately 43 Myr (Eocene), specifically with the ancestor of the recent *Cuapetes nilandensis* (Clade 1). Except a small body size, this species does not show specific morphological adaptations to antipatharian association. The possibly oldest lineage associated with the scleractinian corals forms a common multigeneric composition of *Anapontonia*, *Ischnopontonia*, *Harpilius* and *Philarius* (Clade 4), which was established at approximately 38.2 Myr (Eocene). The genera share some homoplasic adaptations with ectosymbioses, such as strongly hooked dactyli of the ambulatory pereiopods adapted to climbing on coral colonies. An extremely compressed body and similar tail fan structure of the genera *Ischnopontonia* (Fig. 1H) and *Anapontonia* (Fig. 1D) are adaptations to life in narrow spaces amongst corallites of the oculinid coral *Galaxea*^24,30^; the intercorallite channels might be temporarily fully covered by tentacles of exposed polyps. This lifestyle was thus termed ‘semi-endosymbiosis’ by Horká et al.^4^, as potential evolutionary precursors of the true endosymbioses. In contrast, the genera *Philarius* and *Harpilius* have depressed bodies and associate exclusively as regular ectosymbionts with scleractinian corals, mainly of the genera *Acropora* and *Pocillopora*^22^.

A further multispecies symbiotic lineage is represented by the genus *Vir* (Clade 3), whose origin is dated to approximately 21.1 Myr (Miocene). All species of this genus live in associations mainly with the acroporid, pocilloporid and euphylliid genera of scleractinian corals^31,32^. The adaptation to their symbiotic lifestyle is expressed in the loss of the hepatic tooth, partial or full reduction of ambulatory propodal spines, and cryptic colouration, including transparency of the body and appendages^31,33^ (Fig. 1J). Subsequent scleractinian-associated lineages are represented by separate species that appeared in the Miocene (21.9–10.1 Myr), namely: *Eupontonia oahu*, *Cuapetes amymone*, and *C. kororensis*, which live in association with *Pocillopora*, *Acropora*, and *Heliofungia*, and show only minor adaptations to their symbiotic habits, e.g. loss of the hepatic tooth, dense distal setae on the walking propodi, or extremely slender chelae and a specific cryptic colouration, respectively^22,34,35^.

A single crinoid-associated species, *Palaemonella pottsi* (Clade 3), represents the only case of the switch from a free-living lifestyle to the association with echinoderms in the present study group; it originated at approximately 10.4 Myr (Miocene). Retaining the body shape typical for *Palaemonella*^12^, the species also does not show any noticeable morphological adaptation to such a host; its affiliation with the symbiotic life is, however, clearly observed in the deep-red to black cryptic colouration^36^.

In *Palaemonella aliska* (Fig. 1E) and *Eupontonia nudirostris* (Clade 3), a pair of sister-positioned species in the present analyses (Figs. 2, 3), the ability to co-habit with burrowing animals (e.g., alpheids, gobiid fish, or echiurids) had developed. Their type of symbiosis, inquilinism, formed at approximately 14.8 Myr (Miocene). The reduction of the rostrum length, depressed body, stout main chelae in both, and full lack of the epigastric and hepatic teeth in the latter species^15,25^, were evidently due to that mode of life. Inquilinism is best known in the family Alpheidae, in which multiple genera associate with a variety of burrowing animals^37^. In the family Palaemonidae, inquilinism developed only in the Pon-I group, including *Palaemonella shirakawai* (not analysed here)^14^.

As evident from the present and previously published reports^4,5,7,8,10^, the life history of the Pon-I group was largely shaped by coevolution with coral reefs. The coral reefs were deeply impacted by the K–T mass extinction at the end of the Cretaceous, which was one of the most destructive events in the Phanerozoic^38^. However, coral reefs recovered and became increasingly abundant in the Eocene^39^. This also matches the time of either the origin of host associations, or a wider species radiation of the Pon-I group. The first fossil records of the main coral hosts of the present shrimps are dated after the K-T extinction during the Paleogene (e.g., *Euphyllia* 66.0–61.6 Myr, *Acropora* 59.2–56.0 Myr, *Galaxea* and *Pocillopora* 56–33.9 Myr^40^).

The biogeographic history suggested by S-DIVA analysis points to some dispersal and vicariant events shaping the current pattern of the Pon-I group’s distribution. This reconstruction (Fig. 4) estimates the present-day IWP region within the former Paleo-Tethys Ocean as the most likely ancestral area of the present study group, which originated ~ 91.6 Myr (Late to Early Cretaceous). The present shrimp group had radiated across the entire IWP region and subsequently expanded into the Atlantic Ocean. We assume that the spread of the group took place in the following sequence of events: (1) dispersal of *Palaemonella* spp. from the IWP into the eastern Pacific in the Paleocene (∼ 55.2 Myr; *P. asymmetrica* and *P. holmesi*); (2) dispersal into the western Atlantic (2 spp., complex of *“Cuapetes” americanus*) via the eastern Pacific and vicariance event separating the IWP at Eocene (∼ 46.2 Myr). It was the time after the formation of the Eastern Pacific Barrier (EPB), which was considered the largest extension of the open ocean (ca. 5000 km), that separated the IWP area from the eastern Pacific^17^; (3) the another vicariance event, separating the western Atlantic populations from those of the eastern Pacific in the Oligocene (∼ 30.9 Myr), i.e., before the closure of the Isthmus of Panama, followed by a dispersion of *P. atlantica* into the eastern Atlantic in the Miocene (∼ 21.6 Myr). The exact time of the formation of the Isthmus of Panama, which separated the Atlantic from the eastern Pacific and remained isolated from the central Pacific by the EPB, still remains questionable. Bacon et al.^18^ assume that the initial land bridge formed at approximately 23 Myr, and the final closure of the Isthmus of Panama formed between 10 and 6 Myr. Montes et al.^19^ presupposed the earlier formation of the barrier at ∼ 14 Myr, whereas O’Dea et al.^20^ concluded that the potential gene flow continued between the Pacific and Atlantic subpopulations of marine organisms until at least ∼ 2.8 Myr.

The eastern Pacific *Cuapetes canariensis* closely related to IWP *Cuapetes* spp., has been recently described by Fransen et al.^41^, from the Canary Islands. This could indicate alternative dispersal pathways into the Atlantic, as suggested by recent studies^17,42^. The Tethys seaway allowed natural dispersion between the Atlantic and Indian Oceans across the region of the Mediterranean Sea. The closure of this interoceanic seaway at approximately 14 Myr (18–12 Myr) was caused by intense tectonic activity in the Near East^17^. Since the closure of that seaway, remaining possible dispersal to the Atlantic has been limited to the warm-water corridor around the southern tip of Africa, however curtailed by the cold Benguela Current upwelling from the Late Pliocene^43^.

## Conclusions

The current diversity and distribution of palaemonid shrimp have been shaped by a complex interplay of the activity of tectonic plates and climate changes, causing soft and hard barriers. Representatives of the family Palaemonidae, Pon-I group, originated in the IWP as a part of the past Tethys Ocean, most likely dated in the Late Cretaceous, followed by a migration to America via the eastern Pacific before the closure of the Panama Isthmus. Our results suggest that the Pacific population split due to the EPB. It appears that the largest species radiation occurred after the fifth largest extinction (K–T). Ancestral state analysis indicated the formation of eight independent symbiotic lineages of one species, single-generic, or multigeneric, that evolved from free-living ancestors; the oldest associations were with antipatharian (*C. nilandensis*) or scleractinian (multigeneric assemblage of *Philarius*, *Harpilius*, *Ischnopontonia*, *Anapontonia*) corals. Our phylogenetic analyses demonstrate generic and species relationships within the Pon-I group and revealed non-monophyletic nature of the genera *Cuapetes*, *Palaemonella*, *Eupontonia*, and possibly *Madangella*.

## Materials and methods

### Sampling

In this study, 55 analysed specimens belonging to the family Palaemonidae (Supplementary Table S1) were mostly collected during expeditions organised by the National Museum of Natural History, Paris (MNHN), to Papua-New Guinea, New Caledonia and Martinique, by the Australian Institute of Marine Science, Townsville (AIMS), and during field trips organised by Dr. T.-Y. Chan (National Taiwan Ocean University—NTOU, Keelung) and Dr. C.-W. Lin (National Museum of Marine Biodiversity and Aquarium—NMMBA, Kenting) in Taiwan and Dr. N. A. Chadwick (Auburn University, U.S.A.) in Jordan, or the senior author (ZĎ) in Vietnam. Some specimens were obtained on loan from museums along with permission to use them in molecular analyses. Shrimp were collected by standard sampling methods, such as direct hand picking by scuba diving, dredging, and rock brushing, and then preserved in 80% ethanol for deposition in museums. Pieces of tissues were preserved in 96–99% ethanol for subsequent molecular analyses.

### DNA analyses

Genomic DNA was extracted from abdominal muscle tissues, pleopods or eggs using the DNeasy Blood and Tissue Kit or Qiamp DNA Micro Kit (Qiagen, Inc.) following the manufacturer’s protocols. To resolve the phylogenetic relationships, partial mitochondrial 16S rRNA (16S; ∼ 528 bp), the cytochrome c oxidase subunit I mitochondrial DNA (COI; ∼ 658 bp), nuclear 18S rRNA (18S; ∼ 1300 bp), nuclear histone 3 (H3; ∼ 276 bp), phosphopyruvate hydratase (Enol; ∼ 420 bp), and sodium–potassium ATPase α subunit (NaK; ∼ 655 bp) were sequenced. Target gene regions were amplified by polymerase chain reaction (PCR) using primer pairs that can be found in Supplementary Table S2. Polymerase chain reactions, purification and sequencing were conducted following previously described protocols^4^. All sequences were submitted to GenBank (Supplementary Table S1).

### Alignment and phylogenetic analyses

Sequences were aligned using the MUSCLE^44^ algorithm in MEGA vX software^45^. In MEGAX, the protein coding genes (H3, Enol, NaK, COI) were translated to amino acids to control frameshift mutation and stop codons. Substitution saturation of all genes was tested in Dambe v6.4^46^ according to an index by Xia et al.^47^. At the third position of the COI, saturation was detected and thus was excluded from further analyses. GBlocks v0.91b^48^ was used on the individual datasets of genes 16S and 18S to omit highly divergent and poorly aligned regions.

The multigene dataset was concatenated by SequenceMatrix v1.8 software^49^ consisting of 3379 characters. Phylogenies were inferred using both maximum likelihood (ML) and Bayesian inference (BI). The ML analysis was conducted via the web server W-IQ-TREE^50^ (http://iqtree.cibiv.univie.ac.at/) using the best-fit substitution model automatically selected by the software according to the Akaike information criterion (AIC). An ultrafast bootstrap (UFB)^51^ with 10,000 replicates was used in the analysis to assess branch support. The BI analysis was conducted in MrBayes on XSEDE v3.2.7a^52^ via the online CIPRES Science Gateway^53^ using substitution models selected by Partition Finder 2.1.1^54^ (Supplementary Table S3). The Markov chain Monte Carlo (MCMC) algorithm was run for 20 million generations and sampled trees every 500 generations. The convergence of the BI was checked in Tracer v1.6^55^. The phylogenetic trees were displayed in the online application ITOL (Interactive Tree of Life) v6^56^. BI phylogram branch support was assessed by posterior probability (PP), and UFBoot was significant at ≥ 95.

### Divergence time estimation

Divergence dates were inferred using Bayesian relaxed clock uncorrelated lognormal method in the program BEAST v2.6^57^, and as part of the BEAST package, BEAUti 2 created the input file. Molecular clock models were testing in MrBayes v3.2.6 using Bayes factor and a relaxed molecular clock was used (Supplementary Table S4). The optimal substitution models were automatically selected by the package bModel Test^58^ as implemented in BEAUti. The mutation rates are not known for the family Palaemonidae, so the rates for mitochondrial gene were based on the average reported pairwise mutation rates for crustaceans: 0.65% and 0.88% per million years were reported for 16S^59^. The Yule model was set, and the analysis was run for 200 million generations. The reached convergence was checked in Tracer v1.6^55^, and the maximum clade credibility tree was generated in TreeAnnotator v1.10.4^60^ after removing 20% of trees as burn-in. The resulting tree is displayed in FigTree v1.4.3^61^.

### Ancestral analysis

Ancestral reconstruction of the evolution of host associations was implemented in Mesquite v3.5^62^. The analysis was performed using the parsimony criterion based on the BI tree topology. The history of spread of the studied group was estimated using statistical dispersal-vicariance analysis (S-DIVA) implemented in RASP 4.2 software^63,64^. Populations were clustered into four geographical areas. Areas were defined based on known present-day distributions of extant specimens and coded as follows: A—Indo-West Pacific (IWP); B—eastern Pacific (EP); C—western Atlantic (WA); D—eastern Atlantic (EA). Trees generated by BEAST and a consensus tree generated by TreeAnnotator were used as the input. The number of maximum areas was kept at two. GPlates v2.3 software was used to visualise and edit paleogeographic maps^65^.

## Supplementary Information

Supplementary Information.

## Data Availability

The datasets generated and analysed during the current study are available in the ScienceDB repository (Available at: https://www.scidb.cn/en/s/maiii2) and in GenBank database with the accession numbers ON372561-ON372602 for 16S, ON369130-ON369165 for COI, ON376682-ON376720 for H3, ON507934-ON507986 for 18S, ON376592-ON376636 for NaK and ON376637-ON376681 for Enol.
