# Supplementary material for: Molecular phylogeny and historical biogeography of marine palaemonid shrimps (Palaemonidae: Palaemonella–Cuapetes group)
Source: Sci Rep. 2022 Sep 8;12:15237. doi: 10.1038/s41598-022-19372-5 (PMC9458662; doi:10.1038/s41598-022-19372-5)
Supplement: Supplementary file 1 — Supplementary Information. [file 41598_2022_19372_MOESM1_ESM.pdf]

SUPPLEMENTARY INFORMATION

to accompany

Molecular phylogeny and historical biogeography of marine palaemonid shrimps (Palaemonidae: *Palaemonella–Cuapetes* group)

Pavlína Frolová, Ivona Horká, Zdeněk Ďuriš

Supplementary Table S1. List of the specimens and DNA sequences used in the phylogenetic analyses. Used abbreviations and symbols: CBM - Natural History Museum and Institute, Chiba, Japan; EA - eastern Atlantic; EP - eastern Pacific; IWP - Indo-West Pacific; MNHN – National Museum of Natural History, France; MTQ - Museum of Tropical Queensland, Australia; MZUSP - Museu de Zoologia, Universidade de São Paulo, São Paulo, Brazil; NMMBCD - National Museum of Marine Biology and Aquarium (Crustacea Decapoda), Taiwan; NTOU - National Taiwan Ocean University, Taiwan; OUMNH - Oxford University Museum of Natural History, United Kingdom; RMNH - Naturalis Biodiversity Center, Leiden, the Netherlands; UO - University of Ostrava, Czech Republic; WA - western Atlantic; N/A - sequence not available. In brackets the number of species included in the analysis to the total number of species described within the genus is listed

Supplementary Table S2. Primer sequences and PCR conditions used in this study.

Supplementary Table S3. Best-fit substitution models determined with Partition Finder based on the Akaike information criterion and alignment lengths. Models for protein-coding genes (COI, H3, Enol, NaK) are shown for the 1st, 2nd or 3rd codon positions; the saturation test was applied according to Xia et al. (2003).

Supplementary Table S4. **Bayes factor for molecular clock models testing.**

Supplementary Data S5. Details of expeditions.

Supplementary Table S1. List of the specimens and DNA sequences used in the phylogenetic analyses. Newly sequenced data are marked in bold.

Taxon	Region	Sampling location	Host	Voucher number	16S	COI	H3	18S	NaK	Enolase
**Palaemonidae** Rafinesque, 1815										
***Anapontonia* Bruce, 1966 (1/1)**										
A. denticauda Bruce, 1966	IWP	Papua New Guinea	Scleractinia: *Galaxea* sp.	MNHN-IU-2013-11056	MH286364	**ON369130**	MH286367	**ON507934**	**ON376592**	**ON376637**
***Cuapetes* Clark, 1919 (15/30)**										
C. americanus A (Kingsley, 1878)	WA	Martinique	free-living	MNHN-IU-2016-4399	**ON372561**	**ON369131**	**ON376682**	**ON507935**	**ON376593**	**ON376638**
C. americanus B (Kingsley, 1878)	WA	Martinique	free-living	MNHN-IU-2016-7456	**ON372562**	**ON369132**	**ON376683**	**ON507936**	**ON376594**	**ON376639**
C. amymone (de Man, 1902)	IWP	Taiwan	Scleractinia: *Acropora* sp.	NMMBCD5610	KU064812	KU064962	KU065046	**ON507937**	**ON376595**	**ON376640**
C. andamanensis (Kemp, 1922)	IWP	New Caledonia	free-living	MNHN-IU-2018-1170	**ON372563**	**ON369133**	**ON376684**	**ON507938**	**ON376596**	**ON376641**
C. darwiniensis (Bruce, 1987)	IWP	Vietnam	free-living	UO V10-21	**ON372564**	**ON369134**	**ON376685**	**ON507939**	**ON376597**	**ON376642**
C. elegans (Paul'son, 1875)	IWP	New Caledonia	free-living	MNHN-IU-2018-783	**ON372565**	N/A	**ON376686**	**ON507940**	**ON376598**	**ON376643**
C. grandis (Stimpson, 1860)	IWP	Taiwan	free-living	NMMBCD5611	**ON372566**	**ON369135**	**ON376687**	**ON507941**	N/A	N/A
C. ischiospinosus (Bruce, 1991)	IWP	Papua New Guinea	free-living	MNHN-IU-2013-10715	**ON372567**	**ON369164**	**ON376688**	**ON507942**	**ON376599**	**ON376644**
C. johnsoni (Bruce, 1987)	IWP	Vietnam	free-living	UO V12-85A	**ON372568**	N/A	**ON376689**	**ON507943**	**ON376600**	**ON376645**
C. kororensis (Bruce, 1977)	IWP	Australia: Lizard Island	Scleractinia: *Heliofungia actiniformis*	MTQ W-33107	**ON372569**	KU064964	KU065048	**ON507944**	**ON376601**	**ON376646**
C. nilandensis (Borradaile, 1915)	IWP	Taiwan	Antipatharia	NMMBCD5612	**ON372570**	**ON369136**	**ON376690**	**ON507945**	**ON376602**	**ON376647**
C. platycheles (Holthuis, 1952)	IWP	New Caledonia	Scleractinia: *Acropora* sp.	MNHN-IU-2018-353	**ON372571**	N/A	**ON376691**	**ON507946**	**ON376603**	**ON376648**
C. seychellensis (Borradaile, 1915)	IWP	Australia: Lizard Island	free-living	MTQ W-33443	**ON372572**	N/A	**ON376692**	**ON507947**	N/A	**ON376649**
C. longirostris (Borradaile, 1915)	IWP	New Caledonia	free-living	MNHN-IU-2018-509	**ON372573**	**ON369137**	**ON376693**	**ON507948**	**ON376604**	**ON376650**
C. tenuipes (Borradaile, 1898)	IWP	Jordan	free-living	UO Aq09-106	**ON372574**	N/A	**ON376694**	**ON507949**	**ON376605**	N/A
C. uncinatus Bruce, 2012	IWP	Papua New Guinea	free-living	MNHN-IU-2013-11005	**ON372575**	**ON369138**	**ON376695**	**ON507950**	**ON376606**	**ON376651**
***Eupontonia* Bruce, 1971 (2/4)**										
E. nudirostris Marin, 2014	IWP	Solomon Islands	In the hole with Echiura or *Alpheus* sp.	OUMNH-Solom.2016	**ON372576**	**ON369139**	**ON376696**	**ON507951**	**ON376607**	**ON376652**
E. oahu Bruce, 2010	IWP	Marquesas Archipelago	free-living	MNHN-IU-2013-16342	**ON372577**	**ON369140**	N/A	**ON507952**	N/A	N/A
***Exoclimenella* Bruce, 1995 (4/4)**										
E. denticulata (Nobili, 1906)	IWP	Philippines	free-living	NTOU M02460	**ON372578**	**ON369141**	**ON376697**	N/A	N/A	N/A
E. maldivensis Ďuriš & Bruce, 1995	IWP	Australia: Lizard Island	coral rubble	MTQ W-33394	KU064816	KU064968	KU065051	**ON507953**	**ON376608**	**ON376653**
E. sibogae (Holthuis, 1952)	IWP	Australia: Lizard Island	coral rubble	MTQ W-33263	**ON372579**	KU064969	KU065052	**ON507954**	**ON376609**	**ON376654**
E. sudanensis Ďuriš & Bruce, 1995	IWP	Jordan	free-living	UO Aq09-6A	MH286362	KU064970	KU065053	**ON507955**	**ON376610**	**ON376655**
***Harpilius* Dana, 1852 (2/4)**										
H. consobrinus de Man, 1902	IWP	Taiwan	Scleractinia: *Acropora* sp.	NMMBCD5613	**ON372580**	**ON369142**	**ON376698**	**ON507956**	**ON376611**	**ON376656**
H. lutescens Dana, 1852	IWP	Jordan	Scleractinia: *Acropora* sp.	UO Aq09-77	KU064822	KU064976	KU065060	**ON507957**	**ON376612**	**ON376657**
***Ischnopontonia* Bruce, 1966 (1/1)**										
I. lophos (Barnard, 1962)	IWP	Jordan	Scleractinia: *Galaxea* sp.	UO Aq09-103A	**ON372581**	**ON369143**	**ON376699**	**ON507958**	**ON376613**	**ON376658**
***Madangella* Frolova & Ďuriš, 2018 (2/2)**										
M. altirostris Frolova & Ďuriš, 2018	IWP	Papua New Guinea	free-living	MNHN-IU-2015-1581	MH286363	MH286366	MH286369	**ON507959**	**ON376614**	**ON376659**
M. koumacensis Frolova & Ďuriš, 2020	IWP	New Caledonia	free-living	MNHN-IU-2019-2713	MT819437	MT816395	**ON376700**	**ON507960**	**ON376615**	**ON376660**
***Palaemonella* Dana, 1852 (13/23)**										
P. aliska Marin, 2008	IWP	Papua New Guinea	In the hole with gobi fish and *Alpheus* sp.	MNHN-IU-2013-11044	**ON372582**	**ON369144**	**ON376701**	**ON507961**	**ON376616**	N/A
P. asymmetrica Holthuis, 1951	EP	Clipperton Island	free-living	MNHN-IU-2016-8901	**ON372583**	**ON369145**	N/A	N/A	N/A	N/A
P. atlantica Holthuis, 1951	EA	St. Helena	free-living	OUMNH 131244/55/04	**ON372584**	**ON369146**	**ON376702**	**ON507962**	**ON376617**	**ON376661**
P. burnsi Holthuis, 1973	IWP	Hawaii	free-living	RMNH D.28957 **(paratype)**	**ON372585**	**ON369147**	**ON376703**	**ON507963**	N/A	N/A

Supplementary Table S1. List of the specimens and DNA sequences used in the phylogenetic analyses. Newly sequenced data are marked in bold.

Taxon	Region	Sampling location	Host	Voucher number	16S	COI	H3	18S	NaK	Enolase
P. disalvoi Bruce, 1978	IWP	Papua New Guinea	free-living	MNHN-IU-2013-10803	**ON372586**	**ON369148**	**ON376704**	**ON507964**	**ON376618**	**ON376662**
P. hachijo Okuno, 1999	IWP	Papua New Guinea	free-living	MNHN-IU-2015-556	**ON372587**	**ON369149**	**ON376705**	**ON507965**	**ON376619**	**ON376663**
P. holmesi (Nobili, 1907)	EP	Panama: Las Perlas	free-living	MZUSP 33965	**ON372588**	**ON369150**	**ON376706**	**ON507966**	N/A	N/A
P. longidactylus Hayashi, 2009	IWP	Papua New Guinea	free-living	MNHN-IU-2015-1344	**ON372589**	**ON369151**	**ON376707**	**ON507967**	**ON376620**	**ON376664**
P. okunoi Komai & Yamada, 2015	IWP	Japan: Okinawa Island	free-living	CBM-ZC 13082 **(paratype)**	**ON372591**	**ON369152**	**ON376708**	**ON507968**	N/A	N/A
P. pottsi (Borradaile, 1915)	IWP	Taiwan	Crinoidea	NMMBCD5614	**ON372590**	**ON369153**	**ON376709**	**ON507969**	**ON376621**	**ON376665**
P. pusilla Bruce, 1975	IWP	Australia: Lizard Island	free-living	MTQ W-33321	**ON372592**	N/A	**ON376710**	**ON507970**	**ON376622**	**ON376666**
P. rotumana (Borradaile, 1898)	IWP	Australia: Lizard Island	free-living	MTQ W-33176	**ON372593**	**ON369165**	**ON376711**	**ON507971**	**ON376623**	**ON376667**
P. tenuipes Dana, 1852	IWP	Taiwan	free-living	NMMBCD5615	**ON372594**	**ON369154**	**ON376712**	**ON507972**	**ON376624**	**ON376668**
***Periclimenella* Bruce, 1995 (2/3)**										
P. petitthouarsii (Audouin, 1826)	IWP	Jordan	free-living	UO Aq09-94A	**ON372595**	**ON369155**	**ON376713**	**ON507973**	N/A	N/A
P. spinifera (de Man, 1902)	IWP	Vietnam	coral rubble	UO V10-15A	KU064848	KU065007	KU065074	**ON507974**	**ON376625**	**ON376669**
***Philarius* Holthuis, 1952 (5/10)**										
P. albimaculatus Marin & Anker, 2011	IWP	Taiwan	Scleractinia: *Acropora* sp.	NMMBCD5616	**ON372596**	**ON369156**	**ON376714**	**ON507975**	**ON376626**	**ON376670**
P. gerlachei (Nobili, 1905)	IWP	Australia: Lizard Island	Scleractinia: *Acropora* sp.	MTQ W-33364	**ON372597**	**ON369157**	**ON376715**	**ON507976**	**ON376627**	**ON376671**
P. imperialis (Kubo, 1940)	IWP	Taiwan	Scleractinia: *Acropora* sp.	NMMBCD4092	MH286361	**ON369158**	MH286370	**ON507977**	**ON376628**	**ON376672**
P. lifuensis (Borradaile, 1898)	IWP	Taiwan	Scleractinia: *Acropora* sp.	NMMBCD5617	**ON372598**	N/A	**ON376716**	**ON507978**	N/A	**ON376673**
P. rufus Marin & Anker, 2011	IWP	Taiwan	Scleractinia: *Acropora* sp.	NMMBCD5618	**ON372599**	**ON369159**	**ON376717**	**ON507979**	**ON376629**	**ON376674**
***Vir* Holthuis, 1952 (4/6)**										
V. euphyllius Marin & Anker, 2005	IWP	Vietnam	Scleractinia: *Euphyllia* sp.	UO V10-46	KU064864	KU065021	KU065109	**ON507980**	**ON376630**	**ON376675**
V. orientalis (Dana, 1852)	IWP	Australia: Lizard Island	Scleractinia: *Pocillopora* sp.	MTQ W-33130	KU064865	KU065022	KU065110	**ON507981**	**ON376631**	**ON376676**
V. philippinensis Bruce & Svoboda, 1984	IWP	Vietnam	Scleractinia: *Plerogyra* sp.	UO V10-48	KU064866	KU065023	KU065111	**ON507982**	**ON376632**	**ON376677**
V. smiti Fransen & Holthuis, 2007	IWP	Taiwan	Scleractinia: *Physogyra* sp.	NMMBCD5619	**ON372600**	**ON369160**	**ON376718**	**ON507983**	**ON376633**	**ON376678**
**Outgroup**										
***Brachycarpus* Spence Bate, 1888**										
B. biunguiculatus (Lucas, 1846)	IWP	Taiwan	free-living	NMMBCD4093	MH286365	**ON369161**	MH286368	**ON507984**	**ON376634**	**ON376679**
***Leander* Desmarest, 1849**										
L. tenuicornis (Say, 1818 [in Say, 1817-1818])	IWP	Taiwan	free-living	NMMBCD5620	**ON372601**	**ON369162**	**ON376719**	**ON507985**	**ON376635**	**ON376680**
***Palaemon* Weber, 1795**										
P. debilis Dana, 1852	IWP	Taiwan	free-living	NMMBCD5621	**ON372602**	**ON369163**	**ON376720**	**ON507986**	**ON376636**	**ON376681**

Used abbreviations and symbols: CBM - Natural History Museum and Institute, Chiba, Japan; EA - eastern Atlantic; EP - eastern Pacific; IWP - Indo-West Pacific; MNHN - National Museum of Natural History, France; MTQ - Museum of Tropical Queensland, Australia; MZUSP - Museu de Zoologia, Universidade de São Paulo, São Paulo, Brazil; NMMBCD - National Museum of Marine Biology and Aquarium (Crustacea Decapoda), Taiwan; NTOU - National Taiwan Ocean University, Taiwan; OUMNH - Oxford University Museum of Natural History, United Kingdom; RMNH - Naturalis Biodiversity Center, Leiden, the Netherlands; UO - University of Ostrava, Czech Republic; WA - western Atlantic; N/A - sequence not available. In brackets the number of species included in the analysis to the total number of species described within the genus is listed.

Supplementary Table S2. Primer sequences and PCR conditions used in this study.

Marker	Primer name	Primer sequence (5’→3’)	PCR protocol	Primer reference
16S	AR	CGCCTGTTTATCAAAAACAT	Bracken-Grissom et al., 2014	Palumbi, 1991
	1472	AGATAGAAACCAACCTGG		Crandall and Fitzpatric, 1996
COI	LCO1490	GGTCAACAAATCATAAAGATATTGG	5 min at 94 °C, 40x (30 s at 94 °C, 30 s at 48 °C, 1 min at 72 °C), 5 min at 72 °C	Folmer et al., 1994
	Crust F1	TTTTCTACAAATCATAAAGACATTGG		Costa et al., 2007
	HCO2198	TAAACTTCAGGGTGACCAAAAAATCA		Folmer et al., 1994
H3	AF	ATGGCTCGTACCAAGCAGACVGC	Li et al., 2011	Colgan et al., 2008
	AR	ATATCCTTRGGCATRATRGTGAC		Folmer et al., 1994
18S, 1st fragment	2.0	ATGGTTGCAAAGCTGAAAC	2.5 min at 90°C, 10x (50 s at 92°C, 30 s at 49°C, 1 min at 72°C), 36x (30 s at 92°C, 40 s at 49°C, 1 min at 72°C) 3 min at 72°C	Whiting et al., 2002
	9R	GATCCTTCCGCAGGTTCACCTA		
18S, 2nd fragment	1F	CTGGTGCCAGCAGCCGCGGYAA		Machida & Knowlton, 2012
	2RC	TCCGTCAATTYCTTTAAGTT		
Enol	EA2	AGTTGGCTATGCAGGARTTYATGAT	3 min at 94°C, 35x (30 s at 94°C, 1 min at 51°C, 1.5 min at 72°C), 10 min at 72°C	Tsang et al., 2011
	ES2	ACCTGGTCGAATGGRTCYTC		
NaK	for-b	ATGACAGTTGCTCATATGTGGTT	3 min at 94°C, 35x (30 s at 94°C, 1 min at 51°C, 1.5 min at 72°C), 10 min at 72°C	Tsang et al., 2008
	rev	ACCTTGATACCAGCAGATCGGCACTTGGC		

References

Bracken-Grissom, H. D., Robles, R. & Felder, D. L. Molecular phylogenetics of American snapping shrimps allied to Alpheus floridanus Kingsley, 1878 (Crustacea: Decapoda: Alpheidae). *Zootaxa* **3895**, 492–502 (2014).

Colgan, D. J., Hutchings, P. A. & Beacham, E. Multi-gene analyses of the phylogenetic relationships among the Mollusca, Annelida, and Arthropoda. *Zool. Stud.* **47**, 338–351 (2008).

Costa, F. O., deWaard, J. R., Boutillier, J., Ratnasingham, S., Dooh, R. T., Hajibabaei, M. & Hebert, P. D. N. Biological identifications through DNA barcodes: The case of the Crustacea. *Can. J. Fish. Aquat. Sci.* **64**, 272–295 (2007).

Crandall, K. A. & Fitzpatrick, J. F. Crayfish molecular systematics: Using a combination of procedures to estimate phylogeny. *Syst. Biol.* **45**, 1–26 (1996).

Folmer, O., Black, M., Hoeh, W., Lutz, R. & Vrijenhoek, R. DNA primers for amplification of mitochondrial cytochrome c oxidase subunit I from diverse metazoan invertebrates. *Mol. Mar. Biol. Biotechnol.* **3**, 294–299 (1994).

Li, C. P., De Grave, S., Lei, H. C., Chan, T.-Y. & Chu, K. H. Molecular systematics of caridean shrimps based on five nuclear genes: Implications for superfamily classification. *Zool. Anz.* **250**, 270–279 (2011).

Machida, R. J. & Knowlton, N. PCR Primers for Metazoan Nuclear 18S and 28S Ribosomal DNA Sequences. *PLoS ONE* **7**, e46180 (2012).

Palumbi, S. R. *et al.* The simple fool’s guide to PCR (University of Hawaii, 1991).

Tsang, L. M., Ma, K. Y., Ahyong, S. T., Chan, T.-Y. & Chu, K. H. Phylogeny of Decapoda using two nuclear protein-coding genes: Origin and evolution of the Reptantia. *Mol. Phyl. Evol.* **48**, 359–368 (2008).

Tsang, L. M., Chan, T.-Y., Ahyong, S. T. & Chu, K. H. Hermit to king, or hermit to all: multiple transitions to crab -like forms from hermit crab ancestors. *Syst. Biol.* **60**, 616–629 (2011).

Whiting, M. F. Mecoptera is paraphyletic: multiple genes and phylogeny of Mecoptera and Siphonaptera. *Zool. Scr.* **31**, 93–104 (2002).

Supplementary Table S3. Best-fit substitution models determined with Partition Finder based on the Akaike information criterion, and alignment lengths. Models for protein-coding genes (COI, H3, NaK, Enol) are shown for the 1st, 2nd or 3rd codon positions; saturation test was applied according to Xia et al. (2003).

Gene	Substitution models	Length (bp)	GBlock length (bp)
16S	SYM+I+G	528	414
COI	SYM+I+G, SYM+I+G	658	428*
H3	SYM+I, SYM+I+G, SYM+G	276	263
18S	SYM+I+G	1300	1253
NaK	SYM+I+G, SYM+I+G, HKY+G	655	655
Enol	SYM+I+G, HKY+I+G, SYM+I+G	420	366
Total		3837	3379

* saturated; without 3rd codon positions

Supplementary Table S4. Bayes factor for molecular clock models testing.

relaxed clock model:		
run	arithmetic_mean	harmonic_mean
1	-1.999876e+004	-2.006469e+004
2	-1.999546e+004	-2.006375e+004
all	-1.999611e+004	-2.006433e+004

strict clock model:		
run	arithmetic_mean	harmonic_mean
1	-2.006795e+004	-2.013155e+004
2	-2.006969e+004	-2.013144e+004
all	-2.006848e+004	-2.013150e+004

Supplementary Data S5. Details of expeditions.

The PAPUA NIUGINI 2012 expedition was part of the '*Our Planet Reviewed*' project (2016–2019) organized by Muséum National d'Histoire Naturelle (MNHN), Pro Natura International (PNI), Institut de Recherche pour le Développement (IRD), and University of Papua New Guinea (UPNG). The organizers acknowledge funding from the Total Foundation, Prince Albert II of Monaco Foundation, Fondation EDF, Stavros Niarchos Foundation and Entrepose Contracting, and in-kind support from the Divine Word University (DWU). The expedition operated under a permit delivered by the Papua New Guinea Department of Environment and Conservation.
The Kavieng Lagoon Biodiversity Survey (KAVIENG 2014 expedition) was part of the Our Planet Reviewed expeditions organized jointly by Muséum National d'Histoire Naturelle (MNHN), Pro-Natura International (PNI) and Institut de Recherche pour le Développement (IRD), with support from Papua New Guinea's National Fisheries Authority. The organizers acknowledge supporting funding from the Total Foundation, the Laboratoire d'Excellence Diversités Biologiques et Culturelles (LabEx BCDiv, ANR-10-LABX-0003-BCDiv), the Programme Investissement d'Avenir (ANR-11-IDEX-0004-02), the Fonds Pacifique, and CNRS' Institut Ecologie et Environnement (INEE). The expedition was endorsed by the New Ireland Provincial Administration and operated under a Memorandum of Understanding with University of Papua New Guinea (UPNG).
The Madibenthos survey (MADIBENTHOS 2016 expedition) was spearheaded by the French Marine Protected Areas Agency (now part of the French Agency for Biodiversity), the Regional Directorate for the Environment (DEAL), and the Martinique Water Bureau (ODE), with support from the Directorate of the Sea (DM) and the Martinique Natural Regional Park (PNRM). It was implemented by the Muséum national d'Histoire naturelle (MNHN, Principal Investigator Philippe Bouchet), with funding from the European Regional Development Fund (ERDF), the Territorial Collectivity of Martinique (CTM).
The Koumac 2 was part of the *Our Planet Reviewed* expeditions organized by MNHN Paris, in partnership with Conservatoire d'Espaces Naturels (CEN) de Nouvelle-Calédonie. The survey took place in 2018 and 2019, based at Koumac, New Caledonia. The expedition was funded mainly by the Gouvernement de la Nouvelle-Calédonie, Province Nord, Agence Française de la Biodiversité (AFB), the Lounsbery Foundation, Office des Postes et Télécommunications (OPT), Maison de la Nouvelle-Calédonie, and in-kind support from Mairie de Koumac, Société Nationale de Sauvetage en Mer (SNSM), Régiment du Service Militaire Adapté (SMA) de Koumac, Base Navale de Nouméa, AirCalin, Avis Nouvelle-Calédonie, and Socalait. The expedition operated under a permit issued by Direction du Développement Economique et de l'Environnement (DDEE) of Province Nord.
